# In Vitro Recapitulation of Neuropsychiatric Disorders with Pluripotent Stem Cells-Derived Brain Organoids

**DOI:** 10.3390/ijerph182312431

**Published:** 2021-11-26

**Authors:** Maisumu Gulimiheranmu, Shuang Li, Junmei Zhou

**Affiliations:** Department of Central Laboratory, Shanghai Children’s Hospital, Shanghai Jiao Tong University, 1400 Beijing Road West, Shanghai 200040, China; gulmira@sjtu.edu.cn (M.G.); lishuang@shchildren.com.cn (S.L.)

**Keywords:** brain organoid induction, adolescent neuropsychiatric disorders, autism spectrum disorders

## Abstract

Adolescent neuropsychiatric disorders have been recently increasing due to genetic and environmental influences. Abnormal brain development before and after birth contribute to the pathology of neuropsychiatric disorders. However, it is difficult to experimentally investigate because of the complexity of brain and ethical constraints. Recently generated human brain organoids from pluripotent stem cells are considered as a promising in vitro model to recapitulate brain development and diseases. To better understand how brain organoids could be applied to investigate neuropsychiatric disorders, we analyzed the key consideration points, including how to generate brain organoids from pluripotent stem cells, the current application of brain organoids in recapitulating neuropsychiatric disorders and the future perspectives. This review covered what have been achieved on modeling the cellular and neural circuit deficits of neuropsychiatric disorders and those challenges yet to be solved. Together, this review aims to provide a fundamental understanding of how to generate brain organoids to model neuropsychiatric disorders, which will be helpful in improving the mental health of adolescents.

## 1. Introduction

Adolescent neuropsychiatric disorders have been recently increasing, resulting in heavy economic and social burdens. Both genetic and environmental factors contribute simultaneously to the pathology of neuropsychiatric disorders. How to investigate the molecular mechanism underling the abnormal cognitive and behavioral symptoms of neuropsychiatric disorders is still a big challenge due to the complexity of the brain, as well as the ethical constraints of using human brains for research purposes. Due to the species differences between humans and animals, appropriate research models with human genetic backgrounds are needed.

## 2. Emerging Brain Organoids as Research Models of Human Brain Development and Disorders

The early developmental stages of the human brain are complex and patterned in a spatiotemporal manner [1,2]. It’s almost impossible to investigate human early brain development in vivo due to ethical constraints. Molecular and cellular mechanisms regulating human brain development were achieved through previous observational research on rodent and non-primate models. For instance, the primate-specific *TMEM14B*, a marker of basal radial glia, was identified through single-cell transcriptional profiling of sorted human neural progenitor subpopulations [3]. Further research demonstrated that ectopic expression of *TMEM14B* in mouse embryonic neural progenitors could induce postnatal cortical thickening and gyrification [3]. The transcriptome differences between developing mouse and human neocortex were analyzed and 56 genes were identified to be preferentially expressed in humans, among which *ARHGAP11B* displayed the highest degree of radial glia-specific expression [4]. A further functional study using nonhuman primate marmoset demonstrated that human-specific *ARHGAP11B* could increase the number of upper-layer neurons, as well as enlarging the neocortex and inducing folding [5]. Comparing the cortical cell numbers and neuronal cell types across species, including reptiles, birds and mammals also identified large differences on cortical size and cell diversities among species [6]. However, these findings were achieved through either comparing the static samples of humans and animals or performing functional experiments purely through animals. The dynamic developmental process of the human brain could not be experimentally repeated on human embryos. Furthermore, the rodent and non-primate models are limited when recapitulating features of human brain due to the species discrepancy. For example, nuclear distribution factor E-homolog 1 (*NDE1*) is important for human cerebral cortical neurogenesis with different splicing isoforms between human and mouse. Terminal exon 9 of human *NDE1* was absent in mice, which may contribute to different phenotypes of *NDE1* mutations between humans and rodents [7]. Based on these previous reports and considering the ethical constraints, alternative research models with human genomic background in a dynamic system are needed to improve the current knowledge of human brain development and diseases.

Recent research has recapitulated normal and pathological brain conditions with dynamic in vitro culture of brain organoids. Brain organoids are self-organized three-dimensional (3D) structures derived from tissues or human stem cells [8,9,10,11]. Lancaster et al. developed a 3D culture system from human pluripotent stem cells (PSCs), and cerebral organoids were obtained composing various discrete brain regions including the cerebral cortex with various subtypes of neurons. These cerebral organoids could form progenitor zone organization with the abundant outer redial glia stem cells, which is similar to the features of human cortical development [8]. The human midbrain-specific organoids were also cultured in vitro recapitulating the characteristics of midbrain containing spatially organized groups of dopaminergic neurons [9]. The presence of synaptic connections and electrophysiological activities were also observed, as well as the detection of myelination of neurites [9]. Pathological neurodevelopmental diseases and other disorders could also be recapitulated in vitro with 3D brain organoids construction [10,11]. A comprehensive understanding of the current progress and the problems yet to be solved will help to improve the research with this in vitro model.

## 3. Considering the Key Points in the Induction of Brain Organoids In Vitro

### 3.1. Guided Brain Organoids Were Induced Based on the Characters of Neurodevelopmental Patterning In Vivo

In early studies, PSCs were proven to be capable of aggregating into embryonic bodies (EBs) in suspension culture and differentiating into three germ layers in further 2D cell culture conditions [12,13]. Previously, EBs have been induced into the neuronal lineage and formed neural-tube like structures displayed as rosettes in 2D culture. The formation of rosettes recapitulated the in vivo developmental process of radial glial cells [14]. Therefore, understanding the in vivo developmental process of the brain is critical for developing in vitro research strategies of generating 3D brain organoids.

**Figure 1:**
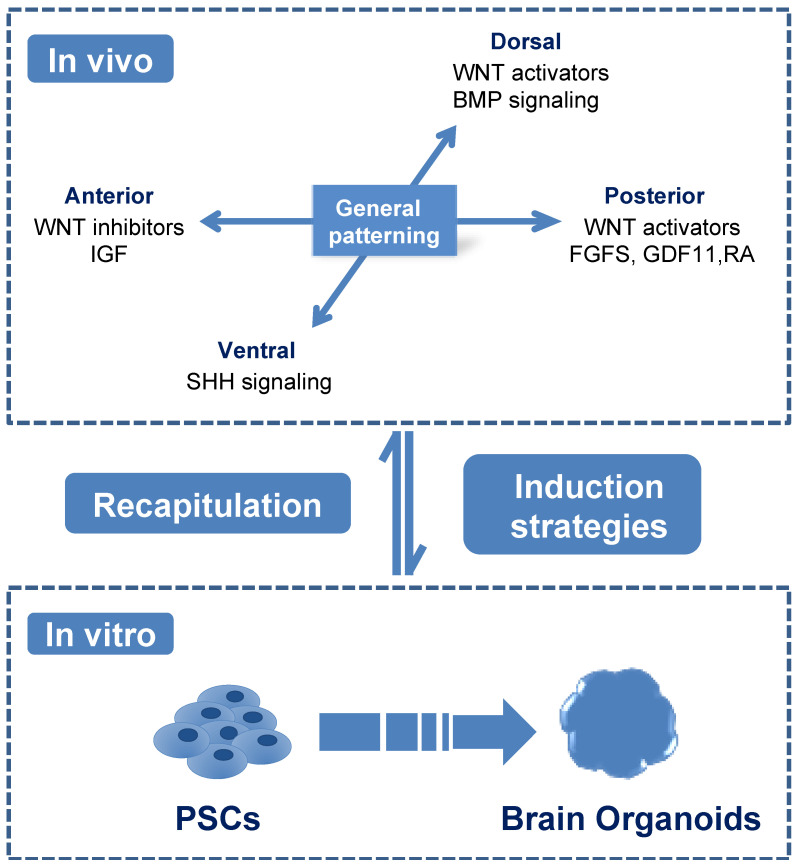
Brain organoids induction in vitro recapitulated the characteristics of human brain in vivo. General patterning principles along the anterior–posterior and dorsal–ventral axis during the in vivo neurodevelopmental process are illustrated in the upper panel. Inductions of brain organoids from PSCs in vitro are illustrated in the lower panel, which followed the general patterning principles in vivo and in turn could be applied to recapitulate the in vivo characteristics of human brain. Abbreviations: PSCs: pluripotent stem cells; IGF: insulin-like growth factors; FGFs: fibroblast growth factors; GDF11: growth differentiation factor11;RA: retinoic acid BMP: bone morphogenetic protein.

The brain consists of a complex neural network, including neurons, astrocytes and oligodendrocytes in different regions [15]. During gastrulation, neural lineage commitment is originated from the ectoderm [15]. Ectoderm cells undergo neurulation and are specified into neuroepithelia cells (NEs) with the inhibition of bone morphogenetic protein pathway by Chordin et al. [16]. NEs generate the neural tube and subsequently begin region specification [14]. Neural regional specification occurs along two axes, the anterior–posterior and dorsal–ventral axes [15] (Figure 1). In the anterior–posterior axes, wingless/integrated (WNT) pathway inhibitors Dickkopf and Frzb, as well as insulin-like growth factors promote forebrain induction, while WNT activators, fibroblast growth factors, growth differentiation factor11 and retinoic acid promote spinal cord formation. In the dorsal–ventral axes, sonic hedgehog pathway (SHH) leads ventralization while as WNT and bone morphogenetic protein pathways favor dorsalization [15]. Besides these extrinsic factors, intrinsic signals from neural progenitors can enrich regionally patterned progenitor subtypes, which could generate various neurons and glia cells. These patterned progenitors often demonstrate a particular transmitter phenotype and generate specified neurons mostly based on their intrinsic signals, such as the progenitors patterned to ventral forebrain would generate more GABA (γ-aminobutyric acid) neurons while dorsal forebrain progenitors are more likely to form glutamate neurons [15]. The further development and maturation of these specified progenitors could be promoted by the presence of neurotrophic factors, such as brain-derived neurotrophic factor and neurotrophic factor 3 [17]. Therefore, understanding the in vivo coordinating signaling pathways properly is critical for recapitulating neural cells of different regional characteristics in vitro.

The brain organoids were first generated through spontaneous differentiation of EBs from PSCs [8]. These in vitro generated brain organoids consisted of broad types of neural cells originated from distinct regions, including forebrain, midbrain, hindbrain, retina and choroid plexus. Therefore, these brain organoids were identified as unguided cerebral organoids. Later studies aimed to achieve region-specific brain organoids in vitro through supplementing subsequent growth factors to the induction medium according to known coordinating signaling pathways in vivo [17,18,19]. Initially, PSCs generated NEs rapidly and efficiently with the presence of dual SMAD inhibition (bone morphogenetic protein pathway inhibitor dorsomorphin and transforming growth factor-β inhibitor SB-431542) [17,18]. Then, NEs were specified into region-specific progenitors at the presence of patterning signals of the anterior–posterior or/and dorsal–ventral axes [18,20,21]. For instance, WNT inhibitors were used to block caudalization and induce forebrain identities [20], while as the addition of SHH agonists together with WNT inhibitors contributed to ventral forebrain identities [18,19]. Other studies indicated that the maintaining of SMAD inhibitors after NEs induction generated a higher yield of organoids with dorsal forebrain identities [17,22]. SHH, fibroblast growth factor-8 and a glycogen synthase kinase-3β inhibitor CHIR99021 were found to promote organoids of midbrain identities [22,23], whereas treatment with fibroblast growth factors guided caudalization [24,25]. These in vitro findings from brain organoids ideally recaptured the in vivo developmental process [15]. Recently, a new study investigated the effects of a signaling center within the brain organoids instead of supplementing extrinsic factors in medium. They embedded SHH-expressing cells at one pole of forebrain organoids and found that these SHH-expressing cells could mimic a developmental organizer and initiate self-organization along two axes [26]. Thus, this asymmetric SHH cue embedding could work as an effective strategy to recapitulate human brain topography, further demonstrating that the strategies of region patterning in vivo could enable more resembled brain organoids in vitro.

Brain organoids have been proven to recapitulate regional properties in vivo [18,27]. Regionally guided brain organoids expressed region-specific markers, such as *NKX2-1* with *FOXG1* in the ventral forebrain and *EMX1* with *FOXG1* in the dorsal forebrain organoids [18]. Morphologically, in vivo brain cortical regions are composed of six distinct layers in which premature neural cells display horizontal migration from inside to outside [8,18]. Among the six layers, the outer subventricular zone is distinguished as the most varied layer between the mouse brain and the human brain. Outer subventricular zone contains outer radial glia cells that display both vertical and horizontal migration to the adjacent region and outer surface [8]. In vitro generated brain organoids also displaying regional characteristics, which expressed *PAX6*, *TBR2* and *MAP2* respectively in the apical surface, adjacent region of apical surface and preplate of organoids reminiscent of radial glia cells in the ventricular zone, intermediate progenitors in the subventricular zone and neurons in the cortical plate in vivo [8,27]. *PAX6* positive RGs were also found to be located outside the ventricular zone of brain organoids which resembled outer glial cells in outer subventricular zone [8]. Besides morphological similarities, gene expressions of brain organoids and fetal brain tissues were also compared using single-cell RNA sequencing and similar transcription profiles were demonstrated between two groups [28]. Furthermore, the physiological properties and functionality of neuronal cells have been proven in studies of PSCs derived brain organoids [17,29]. When exposed to extracellular electrical stimulation, excitatory postsynaptic potentials were detected in brain organoids, which was reminiscent of synaptic responses in the neuronal networks [17]. Current-clamp recordings showed single spikes and burst, which indicated the establishment of complex synaptic events in a neuronal network [17]. Furthermore, electron microscopy imaging confirmed synapse like structures in brain organoids cultured for eight months [29]. These findings indicated that in vitro generated brain organoids recapitulated the characteristics of human brain morphologically and functionally.

### 3.2. 3D Organoid Culture System with or without Scaffolds

The methodology of generating 3D brain organoids is practically different from 2D cell culture. In 2009, researchers generated the first intestinal organoids suspended in a laminin-rich matrigel [30]. Later, this matrigel was applied in other studies to achieve organoids from the brain [8,21], liver [31], kidney [32], etc. Natural or synthetic biomaterials were used to provide spatial supports when generating these 3D organoids [33]. In brain organoids, matrigel has been widely used as a natural biomaterial similar to extracellular matrix, since it contains essential bioactive components for tissue growth in vitro, such as adhesion proteins, collagen, growth factors and metalloproteinases [33]. It also provides scaffolds and tissue-specific biochemical factors through basement membrane ligands, enhances cell attachment and cell–cell interaction, thus providing a dynamic in vitro growth and development microenvironment for organoids [34]. When generating the first brain organoid, EBs being induced for 6 days were embedded into matrigel, expanded into the neural epithelium, and then a fluid-filled cavity was generated [8]. However, some small EBs were observed to fail to form organoids in matrigel. Therefore, researchers used floating fiber microfilament scaffolds before matrigel embedding to increase the surface-area-to-volume ratio [27]. This method increased exposure of matrigel embedded EBs to the neural induction media and promoted the neuroectoderm formation followed by the subsequent cortical differentiation. However, researchers also suspected that matrigels are varied from batch-to-batch and difficult to be specifically designed to meet the requirements of distinct organoids [33]. For these reasons, synthetic materials, such as chemically defined hyaluronan-based hydrogels and polyethylene glycol-based hydrogels were used as alternatives [35]. These synthetic materials could be reproduced with high uniformity and provide desired mechanical properties and degradation rates. Biochemical and biophysical properties of hydrogel formulations have been modulated spatially and temporally to support controlled modifications in organoids [36,37]. Synthetic hydrogel networks could be designed to provide divergent extracellular matrix parameters that govern the separate stages of organoid formation. Poly (lactide-co-glycolide) copolymer fiber microfilaments as floating scaffolds could enhance neuroectoderm formation and cortical tissue characteristics such as polarized cortical plate and radial units [27]. Thus, synthetic materials are considered to reduce batch-to-batch variability and control material properties through chemical and physical modifications [33]. This offers a prospect to artificially designed scaffolds that could meet the desires of distinct tissues.

Organoids could also be generated as 3D spheroids when cultured in proper condition without any scaffolds. Low adherent culture plates were widely used to keep the cell aggregates as spheroids, especially when combined with shaking instruments [38]. PSCs-derived neural progenitor cells could generate 3D cell assembly neurospheres resembling human fetal neural progenitor cells in suspension culture of low adherent culture plates. These neurospheres could generate cortical spheroids and could be cultured up to 20 months [39]. It is more convenient to generate spheroid scaled up to a large volume with defined sizes using high throughput fabrications without any exogenic scaffolds [40]. From this point of view, spheroids without matrigel application may provide a more stable and efficient way to generate organoids for pharmaceutical application, which usually requires a stable high throughput system.

### 3.3. Long Term Maintenance of Human Brain Organoids from PSCs

Since differentiation of neural lineage cells usually takes a relatively long period, in vitro cultivation of brain organoids requires a long-term supply of nutrition and oxygen [8]. Usually, brain organoids have been embedded in matrigel for 4–6 days, then transferred into an agitation device to enhance nutrition and oxygen supply, as well as to exchange wastes [8,22]. Several spinning bioreactors were used as agitation device. In the first study of generating brain organoids in a dynamic system, a spinning flask volume up to 125 mL was used to enhance the medium circulation [8]. In this spinning bioreactor, brain organoids could grow up to a maximum size of 4 mm in diameter by 2 months and could be cultured up to more than one year. However, the substantial volume and bulky size of the spinning bioreactor urged other researchers to design a mini substitute in a 12 well plate, named spinΩ [22]. This mini spinning bioreactor spinΩ is installed on every well of a low-attachment 6-well plate in which the brain organoids were cultured. Since it is installed on every well, it consumes less medium and could guarantee the higher agitation efficiency compared to the spinning bioreactor. However, this highly efficient mini spinning bioreactor was not commercially available since it was made in-house through 3D printing. Consequently, orbital shaker plate, which is more accessible in most labs could be applied as an alternative for either expensive spinning flasks or hard-to-access 3D-printedmini spinning bioreactor [41]. Furthermore, the orbital shaker has been compared with a mini bioreactor in shear stress and fluid flow fields. Results indicated that the orbital shaker plates provided low-shear environment closer to spinning flask [41]. Therefore, an orbital shaker could be suggested as an ordinary agitation device, making the establishment of brain organoids more convenient.

However, even with the agitation device that could speed up the exchange of oxygen and nutrition, a necrosis core still existed in the center of organoids [8,21]. Researchers proposed that the organoids could be sliced to decrease the thickness of organoid, thus enhancing the air–liquid surface and delivering more nutrition to the core area. Then, the brain organoids were sliced with vibratome and further grown in the air–liquid interface [42,43]. Consequently, long and dense axons with specific orientations and improved neuronal survival were detected in the sliced organoids, implying the enhanced nutrition and oxygen diffusion in the sliced organoids [42,43].

In addition to agitating and slicing methods, the introduction of an in vivo–like microenvironment, such as vascular system integration, is supposed to deliver more nutrition and oxygen [44]. Patient induced PSCs derived organoids were co-embedded with endothelial cells differentiated from the same induced PSCs on day 34. After being grown in vitro for an additional 3–5 weeks, the organoids were robustly vascularized and survived two more weeks than non-vascularized organoids [45]. Another study generated functional vasculature-like networks in human brain organoids through ectopically expressing E-twenty six variant2, demonstrating enhanced functional maturation of organoids including increased expression of tight junctions and nutrient transporters in vitro [46]. These vascularized brain organoids were subsequently transplanted into rodent host limbs and perfused blood vessels were found to be connected with the host blood system [46]. Recently, researchers co-cultured endothelial cells from human umbilical vein with dissociated PSCs. These endothelial cells generated a tube-like vascular system and secreted growth factors that could enhance neural differentiation and ameliorate necrosis in the center of organoids [47]. In these studies, vascularization was achieved through in vivo transplantation or in vitro co-culture of endothelial cells. However, it is hard to assure the precise location of vessels through these methods. A recent study applied 3D-printed endothelial cells on geometrically engineered vascular patches to treat ischemia [48], providing the possibilities of applying geometrically engineered patches to enable oriented vascularization in the center of organoids.

Through these methods, brain organoids have been cultured for up to one and a half years in vitro. However, the maximum size appeared at around 2 months and reduced size was observed after 5–6 months after induction [8,49]. It had been estimated that in vivo transplantation of brain organoids into animals would lead to more cell survival and neuronal maturation within the organoids [47,50,51]. Following this, human brain organoids generated in vitro were transplanted into the mouse brain. The transplanted organoids exhibited progressive neuronal maturation and gliogenesis, as well as growing axons to the host brain after transplantation. Results also indicated that the host brain could even receive excitatory inputs when grafted organoids were stimulated [50]. In more recent research, vascularized human brain organoids derived from PSCs were implanted into the mouse cortex. The transplanted organoids were observed to be connected to the mouse blood vessels, establishing functional human–mouse blood vessels in the grafts [47]. These vascularized brain organoids could serve as a paradigm to promote the long-term culture of brain organoids. Meanwhile, ethical concerns about the brain chimeras were also evoked despite the abovementioned achievements of xenograft transplantation [51]. Further research is still needed to enhance long-term culture of human brain organoids, ensuring the advancement of scientific research on revealing the complex human brain development and disorders.

## 4. Recapitulating Neuropsychiatric Disorders with Brain Organoids

Neuropsychiatric disorders are difficult to be experimentally investigated because of the complexity of brain, the inherent species differences between humans and animals in developmental, structural and functional, as well as cognitive aspects. For the heavy economic and social burden resulting from neuropsychiatric disorders, complementary experimental models with human genomic characteristics are urgently needed. Recently developed 3D brain organoids derived from human PSCs provide a promising experimental cellular platform in recapitulating the neuropsychiatric disorders and exploring new therapeutics [52].

**Figure 2:**
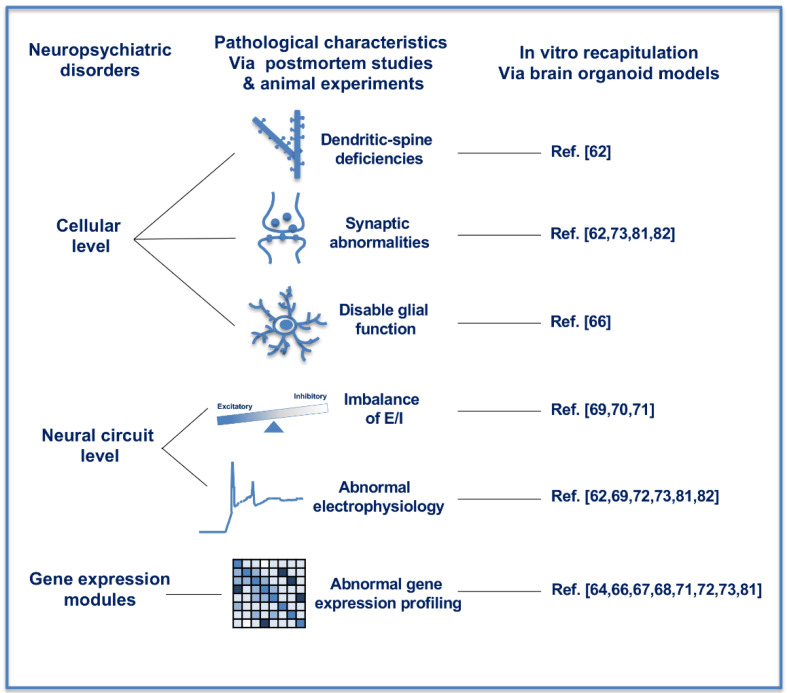
In vitro recapitulation of neuropsychiatric disorders with human brain organoids. The pathological characteristics of neuropsychiatric disorders have been recapitulated in vitro through human brain organoids at the cellular level, neural circuit level and gene expression modules. At the cellular level, in vitro brain organoids exhibited pathological characteristics as dendritic-spine deficiencies, synaptic abnormalities and disable glial functions. At the neural circuit level, brain organoids recaptured the imbalance of excitatory and inhibitory activity and abnormal electrophysiology. Gene expression modules in brain organoids are consistent with those postmortem studies and animal experiments of neuropsychiatric disorders.

The current molecular understanding of cognitive and behavioral symptoms in neuropsychiatric disorders is still superficial because of the structural and functional abnormalities based on the interaction between polygenetic susceptibility and environmental influences [53]. Multiple neural cell types are influenced in the different regions, including the prefrontal cortex, the thalamus, the thalamic reticular nucleus and the basal ganglia in the human brain of neuropsychiatric disorders [53]. Cellular pathological deficiencies and abnormal neural circuit exist broadly in various neuropsychiatric disorders. At the cellular level (Figure 2, left and middle panel), dendritic-spine deficiencies and synaptic abnormalities were reported in multiple neuropsychiatric studies [54,55]. Disable glial function and density abnormalities were also observed in postmortem brain tissues of subjects diagnosed with neuropsychiatric disorders [56,57]. At the neural circuit level (Figure 2, left and middle panel), imbalances in excitatory and inhibitory (E/I) activity are considered as a shared pathophysiological mechanism of neuropsychiatric disorders, including Autism Spectrum Disorders (ASD) and schizophrenia [58]. Imbalance of E/I circuit was observed in the prefrontal cortex of ASD subjects [59]. Through analyzing the cell surface expression of ionotropic glutamate and GABA receptor subunits, mutation of ASD-associated Shank2, which encode the postsynaptic density proteins, resulted in reduced levels of glutamate receptors in the analyzed brain regions, especially in the striatum and thalamus [60]. A more variable functional excitation–inhibition ratio (fE/I) was observed in ASD subjects compared with healthy controls [61]. All of these findings could be recaptured in the dynamic in vitro culture of human brain organoids, demonstrating that human brain organoids derived from PSCs could be a promising model in further research on neuropsychiatric disorders.

So far, the pathological characteristics of neuropsychiatric disorders have been extensively recapitulated in vitro in the research of human brain organoids (Figure 2, right panel). The consistency of in vivo and in vitro cellular results demonstrated the feasibility of the in vitro brain organoids as an experimental model of neuropsychiatric disorders [62,63]. Multi-omics analysis demonstrated that cerebral organoids derived from human PSCs in vitro could recapitulate cerebral cortical development corresponding to the molecular level of before 16 weeks post-conception in vivo [64]. The ASD-associated gene modules through in vitro brain organoid approaches were found to be in significant overlap with previously identified gene modules by differential gene expression between ASD subjects and normal individuals [64]. The in vivo roles of *RAB39b*, a known small GTPase gene associated with X-linked ASD and macrocephaly, were investigated through Rab39b knockout mice and RAB39b-mutated human PSCs [65]. While the impaired cortical neurogenesis, macrocephaly and ASD-like behaviors resembling patients’ clinical phenotypes were observed in knockout mice, human cerebral organoids derived from RAB39b-mutated human PSCs exhibited substantially enlarged capacity due to the increased proliferation and impaired differentiation of neural progenitor cells, both of which through PI3K-AKT-mTOR signaling pathway [66]. CHD8 (chromodomain helicase DNA-binding protein 8) is one of the most commonly mutated genes in patients with ASD, bipolar disorder, schizophrenia and intellectual disabilities. Through RNA-sequencing on CHD8^+/−^ and isogenic control (CHD8^+/+^) cerebral organoids derived from PSCs, differentially expressed genes revealed enrichment of gene modules involved in neurogenesis, neuronal differentiation, forebrain development, Wnt/β-catenin signaling and axonal guidance in CHD8^+/−^ group compared with CHD8^+/+^ group [67,68]. A shift toward GABAergic neurons previously found in the pathology of ASD was also recapitulated with human brain organoids. Accelerated cell cycle and overproduction of GABAergic inhibitory neurons were demonstrated in an ASD-organoid group compared with the control group [69]. Furthermore, *FOXG1* was found to be responsible for the overproduction of GABAergic neurons, as well as positively correlated with the severity of symptoms in ASD subject [69]. E/I imbalance found in neuropsychiatric disorders could also be recapitulated in vitro through neural differentiation of PSCs [70,71]. Psychoses-specific PSCs were induced from somatic cells of three pairs of monozygotic twins with well-controlled genetic background. The iPSCs were neural differentiated and cerebral organoids were consequently constructed. Single-cell RNA-sequencing analysis of the organoids demonstrated excessive GABAergic specification through diminished WNT signaling, resulting in E/I imbalance [71]. Cerebral organoids were generated from iPSCs of individuals diagnosed as schizophrenic and in the healthy control found differentially expressed genes between the two groups involved in nervous system development, synapse function, mitochondrial function, as well as modulation of excitatory and inhibitory pathways. Furthermore, the differentially expressed genes were highly enriched in genes implicated in genome-wide association studies of schizophrenia [72]. Synaptic dysfunction, one of the characteristics in neuropsychiatric disorders, was also demonstrated with human PSCs-derived brain organoids [73,74,75]. Recently published study revealed that brain organoids derived from human PSCs with 16p11.2 copy number variation, the most common copy number variation associated with ASD, demonstrated deficits in neuron migration, ion channel activity, synaptic functions, WNT signaling, etc. through transcriptomic and proteomic profiling [73]. All of these in vitro recapitulations of unbalanced specification of E/I neurons, abnormal synapse function, as well as other pathological findings through organoid investigation, were following that occurred in vivo in numerous psychoses, demonstrating the possibility of investigating the mechanism and developing new therapeutics using this dynamic and easy-to-handle in vitro research model of brain organoid with the human genomic background.

Brain organoids have also recapitulated the molecular progression and pathological characteristics of rare neurodevelopmental disease with psychiatric comorbidities, such as Rett’s syndrome, Fragile X syndrome and Dravet syndrome [74]. These rare neurodevelopmental diseases exhibit varied characteristics and different levels of severity due to genetic and epigenetic anomalies [75]. For example, Rett’s syndrome is mainly caused by mutations in methyl-CpG-binding protein 2 (MECP2) located in the X chromosome [76]. However, the severity and pathological characteristics are varied according to the type and location of the MECP2 mutations [77]. These individual varieties and low prevalence have impeded the research of these diseases. One of the challenges in investigating these diseases is the limited information about the mutation specific neurodevelopmental process. Currently, PSC derived brain organoids brought the chance to investigate the neurodevelopmental progression of rare neurodevelopmental disorders as well as its incompletely characterized pathophysiology. For Rett’s syndrome, brain organoids derived from one female donor carrying the R255X mutation, and one male donor carrying the Q83X mutation, both of which could cause MECP2 mutation, displayed different neurodevelopmental processes and pathology. Organoids from female iPSC line with R255X mutation revealed increased expression of glutamatergic post-mitotic neuronal marker TBR1 and decreased cell proliferation associated with the downregulated intermediate progenitor marker *TBR2*. These organoids also showed decreased synaptogenesis and VGLUT1 puncta density, a specific protein of glutamatergic neurons. However, these results were not observed in the male organoids with Q83X mutant. This indicated that brain organoid is a useful platform for studying the mutation-dependent alterations in Rett’s syndrome [78]. Recently, brain organoids were also evidenced to form complex neural networks modeling Rett’s syndrome in vitro and manifesting the physiologically mechanisms underlying the network abnormalities. In this study, researchers generated cerebral cortex–ganglionic eminence ‘fusion’ organoids to acquire functionally connected excitatory and inhibitory neurons. MECP2 mutant organoids displayed abnormalities in synapse formation revealed by an increased excitatory puncta density and without any significant changes in inhibitory synapses, which was also indicative of the impaired E/I balance. Following tests also demonstrated mutant organoids exhibit hyperexcitability, aberrant neural oscillations and neural network dysfunction evidenced by increased spike frequency and decreased gamma oscillations [79]. Therefore, brain organoid is an applicable platform for studying the neuronal networks of rare neurodevelopmental disease.

However, although promising, challenges still exist with research on neuropsychiatric disorders. Human brain organoids usually have great potential in recapitulating the early developmental stages. But, the majority of neuropsychiatric disorders are associated with synapsis, which is formed in the later stage of human brain development and the symptoms of many neuropsychiatric disorders will deteriorate in later processes [80]. Therefore, more explorations are needed to culture brain organoid to far-more mature stages. The defective interaction between glia-neurons is also relatively hard to be studied in vitro currently [80]. Furthermore, lot variation between brain organoids make it challenging to acquire more reproducible pathology characteristics along different lots [12]. All these concerns are expected to be solved with the progress of new technologies and methodologies.

Besides the challenges in improving the 3D culture system, brain organoids, as an in vitro model, also have some pitfalls in precisely reestablishing the human brain development. Human brain development consists of several different stages related to various cell types. Precisely recapitulating the percentages and interactions within different cell types during in vivo early embryo development is still a significant challenge for brain organoids. Furthermore, human brain development is also associated with its microenvironment, including blood, air and cytokines supply levels. For now, it is difficult to duplicate environmental parameters during brain organoid cultivation. Therefore, more comprehensive and precise investigations are needed to recapitulate the human brain characteristics in vitro.

## 5. Perspectives and Opportunities

Neuropsychiatric disorders are difficult to be investigated due to the complex interaction between polygenic and environmental factors. Proper dynamic research models with human genomic background are important in understanding disease mechanisms and developing effective therapeutics. Although confronting with sorts of technical obstacles, we still can make full use of the human brain organoid model combining with new technologies to investigate the neuropsychiatric disorders. At the beginning stage as PSCs, through combining with CRISPR-mediated genomic engineering, isogenic comparisons of PSCs with various identities caused by psychiatric disorder-associated variants could be generated and these genetically modified PSCs derived brain organoids could be applied to elucidate the detailed mechanism between different variants [81,82]. During cultivation process, artificially designed synthetic materials with more accordant compositions could be used as scaffolds to elevate the reproducibility and generate organoids with standard shapes and sizes [80]. Brain organoids representing different regions could be fused to recapitulate the neuronal connectivity and investigate the mechanisms relates to abnormalities in nerve tracts within different regions [19,79]. Neuronal circuit and network function research methods, such as intracellular whole cell patch-clamp recordings, visualization of calcium transients and high density large extracellular probes, could be used to study the electrophysiological properties of neurons in brain organoids [17,79,83]. Single cell sequencing could analyze the molecular phenotypes at cellular resolution and detect different phenotypes [84]. The advancement of in situ sequencing, which could detect high-throughput spatial gene expression data within and between cells has great potential in elucidating complex cellular interaction and signaling within the 3D dynamic human brain organoids. If there are large genomic changes, the assay for transposase-accessible chromatin by sequencing (ATAC-Seq) could be used to detect the global chromatin accessibility [85]. To assist in drug screening, through combining with high throughput screening of small chemical molecules, possible phenotypic drug screenings could be achieved in a dynamic 3D cellular platform with human genetic background [86]. Together, human brain organoids will enable the investigation of neuropsychiatric disorders in the near future.

## 6. Conclusions

Brain organoids provided a new feasible platform for studying the abnormal neuronal development in adolescent neuropsychiatric disorders as well as for studying the impact of genetic variations on the neuronal development at the cellular and molecular levels. Brain organoids can be induced from human PSCs, thus would eliminate the species differences existed in animal models and the ethical problems in acquiring human embryo brain. In vitro generated brain organoids can be cultured up to 20 months and could be further cultivated with the new technology application. These organoids have been applied in recapitulating the abnormal neuronal development in adolescence neuropsychiatric disorders at the cellular level, neural circuit level and gene expression modules. Thus, it has great potential in investigating the abnormalities and potential treatment of adolescent neuropsychiatric disorders.

## Data Availability

Not applicable.
